# Cardiorespiratory fitness and self-reported physical activity levels of referring mental healthcare professionals, and their attitudes and referral practices related to exercise and somatic care

**DOI:** 10.1192/j.eurpsy.2022.1592

**Published:** 2022-09-01

**Authors:** J. Deenik, L. Koomen, T. Scheewe, F. Van Deursen, W. Cahn

**Affiliations:** 1 Maastricht University, School For Mental Health And Neuroscience, Maastricht, Netherlands; 2 GGz Centraal, Scientific Research, Amersfoort, Netherlands; 3 University Medical Centre Utrecht, Psychiatry, Utrecht, Netherlands; 4 Hogeschool Windesheim, -, Zwolle, Netherlands

**Keywords:** physical activity, exercise, referral practices, Healthcare professionals

## Abstract

**Introduction:**

Physical activity (PA) interventions can improve mental and physical health in people with mental illness, especially when delivered by qualified exercise professionals. Also, the behaviour, engagement and support of referring mental healthcare professionals (HCP) seems essential, but research is scarce.

**Objectives:**

Studying the physical fitness and PA of HCP and the relationship with their attitudes and referral practices related to PA interventions

**Methods:**

HCP at the Dutch Association for Psychiatry conference (2019) were invited to an online questionnaire (demographic/work characteristics, stress, PA levels, knowledge/attitudes regarding PA and referral practices) and cycle ergometer test. Linear and logistic regression were used to study the strongest associations.

**Results:**

115 HCP completed the questionnaire. 40 also completed the ergometer test. 43% (n=50) met the national PA guidelines (≥150min moderate-to-vigorous PA and ≥2x bone/muscle-strengthening exercises a week). Women, HCP in training and HCP with more stress were less active and less likely to meet PA guidelines. HCP with personal experience with an exercise professional were more active and met guidelines more often. Knowledge/attitudes on physical health and PA were positive. Patients were more often referred to PA interventions by HCP who met PA guidelines (OR=2.56, 95%BI=0.85–7.13) or had higher beliefs that exercise professionals can increase adherence to PA interventions (OR=3.72, 95%BI=1.52–9.14).

**Conclusions:**

It’s positive that HCP report importance and relevance of PA in mental healthcare. Although there is strong evidence for PA interventions in the treatment of people with mental illness, referral to such interventions can partly depend on the PA behaviour and attitude of patients’ physician/clinician.

**Disclosure:**

No significant relationships.

